# Competences of Paramedics in Prehospital Emergency Care: An Umbrella Review

**DOI:** 10.1155/emmi/5400734

**Published:** 2026-11-15

**Authors:** Marko Leinonen, Petri Roivainen, Anna-Maria Tuomikoski, Merja Sjöman, Kristina Mikkonen

**Affiliations:** ^1^ Research Unit of Health Sciences and Technology University of Oulu Oulu Finland oulu.fi; ^2^ School of Health and Social Care Oulu University of Applied Sciences (OUAS) Oulu Finland; ^3^ North Ostrobothnia Wellbeing Services County Oulu University Hospital Oulu Finland ppshp.fi; ^4^ Medical Research Center Oulu Oulu University Hospital and University of Oulu Oulu Finland oulu.fi

**Keywords:** competence, paramedics, prehospital emergency care, umbrella review

## Abstract

**Aims:**

The aim of this umbrella review is to describe and define the competences required of paramedics in out‐of‐hospital emergency medical care.

**Design:**

The review was conducted in accordance with the methodological guidelines of the Joanna Briggs Institute.

**Data sources:**

Keywords were defined according to the PCC‐based inclusion and exclusion criteria. Data were retrieved from the Medic, PubMed, Scopus, CINAHL and ProQuest databases. Publications in Finnish and English were included. The search was conducted in 2024.

**Review Methods:**

Five reviewers independently screened the eligibility of reviews based on titles and abstracts (*n* = 2224) and full texts (*n* = 105). In the final stage, 20 studies were selected and critically appraised. Methodological quality was assessed using the JBI critical appraisal checklist. Data were analysed using inductive content analysis. Confidence in the synthesised findings was assessed using the GRADE‐CERQual approach.

**Results:**

Eight reviews were included in the final synthesis. The competences required in out‐of‐hospital emergency medical care were categorised into five main domains: interpersonal and teamwork competence; cognitive and decision‐making competence; professionalism and ethical competence; clinical competence; and competence across paramedic certification levels and in special or high‐stress situations.

**Conclusion:**

This umbrella review provides a comprehensive synthesis of the competences required of paramedics in out‐of‐hospital emergency medical care, both currently and in the future. Furthermore, future research should examine which educational and assessment approaches most effectively support the ongoing maintenance and development of competence within a changing operational context and an expanding scope of practice.

**Summary**
• What is already known.
◦ Out‐of‐hospital emergency medical care has undergone substantial change due to health service centralisation, technological advancement, population ageing and increasing service demand. As a result, the competences required of paramedics have expanded and become more complex, with contemporary paramedic practice demanding comprehensive capabilities to manage both urgent and nonurgent care needs.
◦ Although paramedic competence has been examined in numerous previous studies, the focus has largely remained on isolated competence domains. Consequently, existing research has not provided a comprehensive understanding of the breadth and multidimensional nature of the competences required of paramedics.
• What this paper adds.
◦ This review shows that the competences required of paramedics in out‐of‐hospital emergency medical care are extensive, encompassing interpersonal and teamwork competence, cognitive and decision‐making competence, professionalism and ethical competence, clinical competence, as well as competence across different certification levels and in special situations.
◦ Furthermore, the review synthesises the identified competence areas into a coherent framework that delineates the scope of competences required in out‐of‐hospital emergency medical care and provides a clear basis for examining both current and future competence needs.

## 1. Introduction

Over recent decades, the operational environment of prehospital emergency care has undergone significant change. These changes have been driven primarily by population ageing, increasing demand for health services and technological advancement [1–3]. According to the World Health Organization [4], the proportion of people aged 60 years and older worldwide is expected to double by 2050. In the United Kingdom, the United States and Finland, the proportion of the population aged 65 years and older is projected to increase to approximately one‐quarter over the coming decades. In particular, the relative share of the oldest age groups within the population is expected to increase substantially [5–7].

The content of prehospital emergency medical services has also changed qualitatively [8], with a key developmental trend being an increase in the number of patients requiring nonurgent care [9, 10]. This global phenomenon is also evident in Finland, as reflected in an increasing number of nonurgent calls to emergency dispatch centres [11].

These changes have expanded the role of paramedics and diversified their competence requirements.

The effects of these changes are particularly evident in the expansion of paramedics’ scope of practice and the increasing clinical responsibility they assume. In the United Kingdom, the role of paramedics has been developed toward more autonomous decision‐making and prescribing authority [12], whereas in Canada, paramedics are required to demonstrate competence in supporting people with long‐term conditions to live at home and in complementing health and social care services [13].

In Finland, paramedic practice requires broad professional competence and an autonomous approach to work, and the management of emergency patients is of a high standard in international comparison [14]. This is particularly evident in emerging models of care, such as mobile hospital models [15] and community paramedicine [16]. In the future, competence requirements are expected to increasingly emphasise the recognition and management of acute phases of long‐term conditions [10], as well as broad‐based collaborative capabilities with security and national defence actors [17, 18].

Paramedic competence has been described in several international competency frameworks, including the U.S. National EMS Education Standards (National Highway Traffic Safety Administration [19], the UK Standards of Proficiency: Paramedics [20], the Australian Professional Capabilities for Registered Paramedics framework [21] and the European EPaCur framework [22]. These frameworks describe key areas of paramedic competence, including clinical competence, communication competence, safety and risk management competence and decision‐making competence.

The aim of this umbrella review was to describe and systematically structure the competences required of paramedics in out‐of‐hospital emergency medical care.

### 1.1. Background

Traditionally, the primary role of prehospital emergency care has been the urgent treatment of acutely ill and injured patients and, when necessary, their transport to further care. The work also includes assessing care needs among nonurgent patients, initiating immediate treatment and planning further care in collaboration with patients, relatives and relevant stakeholders [23–25]. The planning and delivery of prehospital emergency services require close collaboration with emergency healthcare facilities [26].

Prehospital emergency care is delivered across a wide range of operational environments, including private homes and public settings, regardless of the time of day, weather conditions, or other environmental factors. The work often entails risks and uncertainties associated with these conditions [27]. Prehospital emergency care tasks range from life‐threatening emergencies and major incidents to sudden illnesses, violent situations, mental health disorders, functional decline among older adults, exacerbations of chronic conditions and social emergencies [28]. The assessment of nonurgent prehospital patients conducted by emergency dispatch operators is complicated by the fact that patients’ symptoms are often mild and atypical and are rarely attributable to an acute medical cause [29–31]. Distinguishing between physical and social symptoms is often challenging, which further complicates the assessment of patients’ care needs [32].

It is estimated that approximately one‐fifth of patients suffer from long‐term chronic conditions, such as pain‐related symptoms, and that a substantial proportion of these patients repeatedly use prehospital emergency services [10, 30, 32]. Patients’ symptoms may also be related to social problems, uncertainty regarding self‐care or difficulties in managing everyday life. Often, patients do not recognise the urgency or nature of their symptoms or situation, and this may not be adequately conveyed to emergency dispatch operators during the call [33–35]. Problematic alcohol use is also frequently present as an underlying contributing factor [36]. In addition, emergency calls are repeatedly initiated from hospital wards and residential care facilities to prehospital emergency units, for example, to obtain lifting assistance or to support the assessment of care needs [37, 38]. As a result, tasks are frequently directed to prehospital emergency services [34, 35]. Due to the diversity and complexity of these situations, paramedics are required to possess a broad knowledge base, as well as versatile skills and the capacity to act flexibly, in order to provide appropriate and safe care that responds to patients’ individual needs in continuously changing and multifaceted situations [39].

Competence refers to an individual’s knowledge, skills, attitudes, values and attributes that enable them to perform tasks in accordance with expectations and to achieve defined goals [40]. In this review, competence is understood as the broader, integrated capability to perform safely, effectively and professionally in paramedic practice, and it includes the knowledge, skills and attitudes required to meet the demands of professional practice. By contrast, competency refers to a specific, observable and assessable component of practice, such as an individual skill, behaviour, or performance outcome. Accordingly, the term competence is used throughout this manuscript when referring to the overall professional capability required of paramedics, whereas competency is used only when referring to discrete elements within competency frameworks or when retained in the official title of a framework. Nursing competence, which is required to fulfil professional nursing responsibilities, is formed by an individual’s ability to integrate and apply knowledge and skills across diverse situations, as well as by their individual attributes [41].

In the United Kingdom, nursing education, competence and the right to practise are regulated by the Nursing & Midwifery Council [42], which is responsible for approving education programmes, defining competence requirements and maintaining the register of registered nurses. Education leading to qualification as a nurse is provided by higher education institutions and lasts 3 years (360 CATS) [43]. Nursing competence is defined as safe, effective and professionally accountable practice that integrates evidence‐based knowledge, clinical skills and professional values [44]. In Norway, nursing education, competence and the right to practise are governed by national legislation, and the authorisation of healthcare professionals is the responsibility of the Norwegian Directorate of Health [45]. Education leading to qualification as a nurse is provided by higher education institutions and lasts 3 years (180 ECTS). Nursing competence is defined as evidence‐based, patient‐centred and ethically responsible practice that requires clinical decision‐making capability and readiness to work in interprofessional and culturally diverse environments. Furthermore, competence definitions emphasise health promotion, disease prevention, safeguarding patient safety and continuous professional development [46].

In Finland, nursing education must comply with the requirements of the revised Professional Qualifications Directive of the European Parliament and of the Council [47] and with national legislation governing the recognition of professional qualifications [48]. The nursing degree is a university of applied sciences degree with a duration of 3.5 years (210 credits), exceeding the minimum required under EU legislation by 30 credits [49, 50]. The minimum professional competence of nurses consists of nine core competence areas, including client‐centredness, ethical and professional nursing practice, leadership and entrepreneurship competence, clinical nursing, evidence‐based practice and decision‐making, guidance and teaching competence, the promotion of health and functional capacity, knowledge of the social and healthcare operating environment and the quality and safety of social and healthcare services [51].

Internationally, paramedic competence has not been defined as consistently as nursing competence, which reflects substantial variation in educational pathways and healthcare system structures across countries. However, paramedic competence has been outlined in recommendations issued by several international organisations and in research within the field [52].

In the United States, education for prehospital emergency care personnel is guided by federal recommendations and state‐level legislation. The National EMS Scope of Practice Model defines the roles, responsibilities and limits of practice associated with different levels of EMS education, whereas the National EMS Education Standards define the minimum educational content and competence requirements [19, 53]. The duration and structure of education at the Emergency Medical Technician (EMT) and Advanced Emergency Medical Technician (AEMT) levels vary across states and education providers. Education leading to paramedic qualification follows two main pathways: certificate programmes typically last 6–12 months, whereas Associate Degree programmes last 1–2 years, and both pathways usually require EMT‐level certification [19].

In the United Kingdom, paramedic education and the right to practise are regulated by the Health and Care Professions Council (HCPC). The HCPC operates under the Health Professions Order 2001 and is responsible for the registration of paramedics and for regulating professional competence [54]. EMT education is provided by NHS Ambulance Trusts and private education providers, and the training duration at London Ambulance Service is 15 months [55]. University‐based education leading to qualification as a paramedic includes the BSc Paramedic Science and BSc Paramedic Practice (Hons) degrees, which typically last approximately 3 years. In addition, the Paramedic Degree Apprenticeship is a work‐based training programme lasting 3–4 years and leading to a BSc Paramedic Science degree [56].

In Finland, the definition of healthcare professionals’ competence is based on national legislation and nationwide educational steering. The Decree of the Ministry of Social Affairs and Health on Emergency Medical Services (585/2017) specifies the qualifications required of personnel working in prehospital emergency care units and the minimum educational requirements. At vocational education institutions, it is possible to complete basic qualifications in social and healthcare services and in the rescue field, with a primary focus on basic‐level prehospital emergency care. These qualifications include the rescue worker qualification (90 credits), the practical nurse qualification with a specialisation in prehospital emergency care (180 competence points), the basic‐level paramedic qualification (180 competence points) and the nursing degree (210 credits).

The University of Applied Sciences degree in paramedicine has a scope of 240 credits and provides direct eligibility to practise as an advanced‐level paramedic [57]. According to a government proposal concerning paramedic education, holders of a nursing degree (210 credits) may qualify as paramedics by completing a separate paramedic education programme with a scope of 90 credits. Paramedics graduating from universities of applied sciences are currently licensed in Finland only as nurses [58].

In Finland, the national network of university of applied sciences educators in paramedicine defines the core competence areas of paramedics as out‐of‐hospital emergency care for acutely ill and injured patients, assessment of care needs, guidance and nonurgent prehospital care, operational leadership and professional practice within the paramedic work community, cooperation with authorities and other stakeholders, preparedness and readiness as part of comprehensive security, and special situations in prehospital emergency care and acute nursing. These core competence areas have also been incorporated into the draft of a new decree on paramedic education, which is currently in the final stage of the consultation process [58].

In international and national research, competence required in out‐of‐hospital emergency medical care has predominantly been examined within narrowly defined domains, resulting in the lack of a comprehensive and systematically structured understanding of the required competence. International research has focused primarily on qualifications required in paramedic practice [59, 60], competence assessment [61], instruments for measuring competence [52] and structures for competence management and registration [62]. Within nursing science, competence has been examined from perspectives including professional nursing competence [63], clinical decision‐making [64], paediatric nursing [65] and intensive care nursing [66]. This is noteworthy, as healthcare professionals’ competence has been shown to be associated with quality of care, critical thinking, patient safety and professional commitment [67–69]. This review provides new knowledge on the competence required of paramedics in out‐of‐hospital emergency medical care and complements existing research on the topic.

## 2. The Review

### 2.1. Aims

The aim of this umbrella review was to describe and systematically organise the competences required of paramedics in out‐of‐hospital emergency medical care based on prior research evidence.

The research question was: What competences are required in prehospital emergency care now and in the future according to previous research?

### 2.2. Research Design

This umbrella review was undertaken to identify and synthesise existing research evidence on the competences required of paramedics in out‐of‐hospital emergency medical care [70]. The review followed methodological guidance from the Joanna Briggs Institute (JBI) [71], and reporting adhered to the PRISMA Statement, which promotes transparent and comprehensive reporting of systematic reviews and meta‐analyses [72]. The review protocol was registered in the PROSPERO database (CRD420251029207).

### 2.3. Search Methods

The umbrella review was conducted in September 2024 across five electronic databases: PubMed, CINAHL, Medic, Scopus and ProQuest. Study selection was guided by inclusion and exclusion criteria derived from the research question and structured according to the PCC framework as follows: Participant = prehospital emergency care professionals; Concept = competence in prehospital emergency care; Context = out‐of‐hospital emergency medical care [70]. In addition, the inclusion criteria required that eligible publications were review articles. The exclusion criteria were: (1) studies involving educators, teachers, lecturers, researchers, supervisors, students, or participants other than prehospital emergency care professionals; (2) original research articles; (3) non‐peer‐reviewed publications; (4) articles published before 2004 were excluded from the review to ensure that the reviewed material reflects the competence currently required in out‐of‐hospital emergency medical care, as competence requirements have evolved substantially over the past 2 decades and (5) publications in languages other than Finnish or English. The search strategy was developed in collaboration with an information specialist and tailored to each database. Search strings were constructed by combining search terms using the Boolean operators AND, OR and NOT (Supporting file 1).

### 2.4. Search Outcomes

The database searches identified 3342 records. After the removal of duplicates (*n* = 1118), 2224 records were screened based on their titles and abstracts and 2119 were excluded. A total of 105 full‐text reports were sought for retrieval. Of these, 67 were excluded based on the exclusion criteria or because the full text was unavailable. The remaining 38 reports were assessed for eligibility. Further 18 reports were excluded because they were not systematic, scoping or integrative reviews.

Twenty reviews underwent critical appraisal. Following the critical appraisal, 12 reviews were excluded. Eight reviews were included in the final synthesis (Figure 1).

**FIGURE 1:**
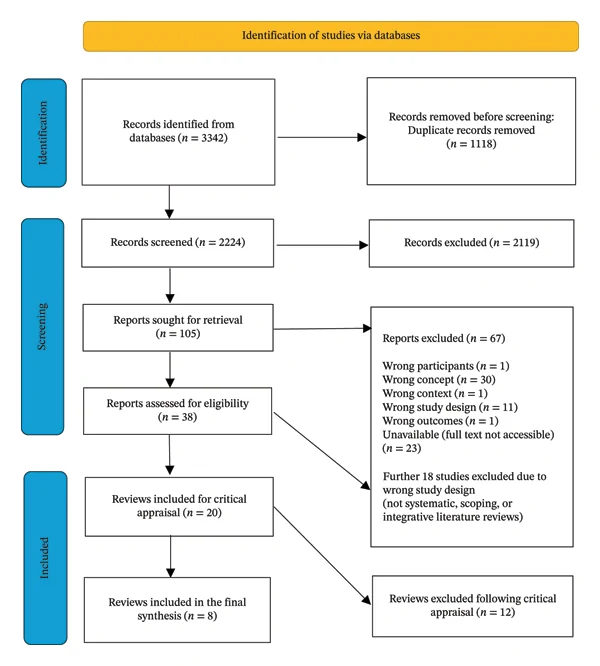
PRISMA flow diagram [72].

### 2.5. Quality Appraisal

The methodological quality of the 20 reviews selected for critical appraisal was independently assessed by five reviewers using the JBI critical appraisal checklist for systematic reviews [70]. The appraisal focused on the reliability, methodological quality and credibility of the reviews, using 11 predefined criteria. Each criterion was rated as ‘yes’, ‘no’, ‘unclear’ or ‘not applicable’, and one point was assigned for each ‘yes’ response. Any disagreements between the reviewers were resolved through discussion, and no external reviewer was involved. The results of the quality appraisal were as follows: one study achieved the maximum score of 100% [73], one study scored 90% [74], four studies scored 80% [75–78], one study scored 60% [79] and one study scored 50% [80] (see scores in Supporting file 2).

### 2.6. Data Extraction and Synthesis

Key data relevant to the research question were extracted systematically in order to minimise bias [81]. From the included review articles, the following information was collected: authors, year of publication, country of origin, title of the study, type and purpose of the review, data collection and analysis methods, participants, key concept, context and main findings related to the competence required in out‐of‐hospital emergency medical care (see Table 1). The data were analysed using inductive content analysis to structure research findings describing the competences required of paramedics working in out‐of‐hospital emergency medical care [82]. At the initial stage of the analysis, the original text was divided into meaning units (single words, expressions or larger units) according to the research question. A total of 73 research‐question‐related codes were generated from the meaning units. In the subsequent stage, 71 subcategories, 16 categories and 5 main categories were identified (see Table 2).

**TABLE 1:** Data extraction.

Author, country	Review type	Purpose	Methodology	Participants	Concept	Context	Key findings
Abelsson et al., [74], Sweden	Integrative literature review	To describe what ambulance nurses and paramedics perceive as important for learning in simulation exercises	CINAHL, PubMed and Scopus studies = 7 year of publication: 2001–2014 Narrative and thematic synthesis	EMTs, Medics (basic, intermediate and paramedic levels), other healthcare professionals (physicians, nurses, respiratory therapists, physician assistant) and students from nursing specialties (*n* = 662)	Technical, nontechnical, cognitive and reflective competence	Prehospital emergency care	Simulation‐based training in prehospital care enhances technical competence, such as managing life‐saving interventions under pressure. Nontechnical competence, including effective communication, teamwork and decision‐making, is developed through realistic scenarios. Simulation promotes cognitive competence, such as clinical reasoning, situational judgement and adaptability to rapidly changing circumstances. Structured debriefing sessions foster reflective competence, enabling self‐assessment, reinforcement of skills and experiential learning. These competencies are essential for managing complex and stressful prehospital situations, ensuring safe and high‐quality patient care
Anderson et al. [76], New Zealand	Systematic review	To explore how ambulance personnel are prepared and supported to withhold or terminate resuscitation and manage patient death	Medline, CINAHL, ScienceDirect, PsycINFO Studies = 16 year of publication: 2000–2018 Scoping review using Arksey & O’Malley’s framework with narrative synthesis	Ambulance personnel (*n* = 3623)	Ethical, emotional, communicative and decision‐making competence in managing the termination of resuscitation and patient death in prehospital care	Prehospital emergency care	Ambulance personnel require ethical decision‐making, emotional regulation and communication competence to manage resuscitation termination and patient death. Key competence needs include delivering death notifications, managing moral distress and applying ethical judgement in unpredictable prehospital settings. Reflective competence and access to structured support (e.g. debriefing) are essential to maintain professional performance.
Alruwaili et al., [73], Saudi Arabia	Scoping review	To examine the role and outcomes of prehospital intensive care units in trauma care	PubMed, Scopus, CINAHL and Web of Science Studies = 13 Year of publication: 2012–2023 Scoping review following PRISMA‐ScR guidelines	Trauma patients treated by PICUs	Clinical competence, advanced life support skills, specialised training, prehospital critical care	Prehospital emergency care	Prehospital intensive care units (PICUs) demand high levels of clinical competence, highlighting the need for specialised skills in managing critically ill patients. These include airway management, resuscitation and the execution of complex interventions. Personnel working in PICUs receive specialised training and must be authorised and pass regular skill reaccreditation. Most life‐saving interventions performed in the prehospital environment exceed the competence level of paramedics or basic‐level prehospital personnel. The developing field of PICUs shows significant disparities in standardisation, and universally accepted guidelines governing their design, staffing, usage and proliferation are lacking
Cormack et al. [79], United Kingdom	Scoping review	To identify which nontechnical skills are associated with ad hoc teams managing out‐of‐hospital cardiac arrest and to map the existing literature on this topic	MEDLINE, AMED, CINAHL, PsycINFO, PsycARTICLES and ScienceDirect = 12 Year of publication: 2000–2020 Scoping review using Arksey & O’Malley’s framework with thematic analysis	Paramedics, ambulance clinicians, emergency medical technicians and other healthcare professionals involved in OHCA management	Leadership, communication, teamwork, situational awareness and decision‐making as nontechnical skills in prehospital cardiac arrest care	Prehospital emergency care	Clear leadership during out‐of‐hospital cardiac arrest improves team coordination, protocol adherence and role clarity. Structured communication, especially closed‐loop, enhances information flow and reduces errors. Adaptive teamwork supports cohesive performance and patient safety under pressure. Situational awareness enables resource management and control in dynamic environments. Effective decision‐making is critical in managing uncertainty and making timely clinical judgements. Simulation and debriefing strengthen nontechnical skills, building confidence, reflection and readiness
Evans et al. [78], Canada	Scoping review	To examine how nontechnical skills are defined, measured and applied in ad hoc resuscitation teams in prehospital, emergency and trauma settings	EMBASE, CINAHL, MEDLINE, PsychINFO Studies = 61 year of publication: 1992–2021 Scoping review using PRISMA‐ScR and Arksey & O’Malley’s framework with descriptive extraction, coherence analysis and citation network analysis	Physicians, nurses, paramedics, respiratory therapists, prehospital responders and members of intersectoral teams such as paramedics and police	Nontechnical competences including leadership, structured communication, adaptive teamwork, situational awareness and critical decision‐making in prehospital cardiac arrest care	Prehospital emergency care	Nontechnical competences such as leadership, communication, teamwork, situational awareness and decision‐making are essential for effective performance in ad hoc resuscitation teams. Leadership supports coordination, prioritisation and shared mental models that enhance team responsiveness and cohesion. Structured communication enables information clarity, reduces uncertainty and facilitates faster clinical decision‐making in time‐critical scenarios. Adaptive teamwork helps redistribute responsibilities dynamically, ensuring flexibility and problem‐solving under pressure. Situational awareness allows for early recognition of risks and supports appropriate, context‐sensitive actions. The inconsistent definitions and variable use of measurement tools for nontechnical skills across studies demonstrate the need for a unified competence framework to guide training, evaluation and future research on team performance in emergency care.
Evans et al. [75], United Kingdom	Systematic review	To identify international evidence on extended skills training for paramedics beyond baseline competencies and assess its impact on patient care and service use	Medline, EMBASE, CINAHL, ProQuest, Scopus, Cochrane, York CRD, NHS Evidence, Department of Health, UK Public Health Observatory, British Library, Web of Science, Policy Network, Zetoc, Newcastle University Library; Studies = 19 Year of publication: 2001–2014 Narrative and thematic synthesis	Paramedics, paramedic practitioners, community paramedics, EMS personnel and paramedic students	Skills beyond baseline competencies, including history taking, examination, diagnostics, interventions, teamwork and decision‐making	Prehospital emergency care	Extended paramedic roles strengthen clinical competence, such as independently assessing and managing elderly patients with minor conditions in the community. Decision‐making competence is demonstrated through accurate triage, safe discharge and appropriate referral to healthcare services. Collaborative competence is developed by working with general practitioners and integrating into multidisciplinary care teams. Preventive competence includes conducting falls risk assessments, reviewing vaccination status and providing basic health advice. Environmental safety competence involves identifying home hazards and offering safety guidance during patient visits. Public health competence emerges through participation in community outreach, health education and rural service development. These competencies are essential for delivering patient‐centred care, reducing unnecessary hospital admissions and supporting sustainable prehospital systems
Gent [80], Australia	Systematic Literature Review	To identify what is known about continuing professional development (CPD) for paramedics	ERIC, MEDLINE Complete and CINAHL; Studies = 8 Year of publication: 2000–2015 Integrative review with narrative and thematic synthesis	Paramedics, ambulance clinicians and emergency medical technicians involved in continuing professional development in prehospital care (*n* = 2773)	Clinical competence, nontechnical competence, cognitive competence and reflective competence	Prehospital emergency care	Continuing professional development (CPD) in prehospital care supports clinical competence, including the performance of procedures such as airway management, intravenous access and pain management in real patient encounters. Nontechnical competence, such as confidence, ethical commitment and decision‐making, is strengthened through experiential learning and reflective practice. CPD enhances cognitive competence by improving theoretical knowledge, clinical understanding and documentation skills. Reflective competence is fostered by promoting self‐directed learning, professional responsibility and engagement with personal development. Together, these competencies are vital for maintaining high standards of care in unpredictable and high‐pressure prehospital environments
Horrocks et al. [77], Australia	Scoping Review	To identify the most common core competency domains for responding paramedics in disaster management	PubMed, MEDLINE, Science Direct, Scopus, Embase and ERIC Studies = 6 Year of publication: 2000–2017	Paramedics and emergency medical technicians involved in disaster response and disaster health management	Core competence, technical/clinical competence and specialist technical/clinical competence	Prehospital emergency care	Core competencies enable paramedics to effectively manage disaster situations through communication, triage, ethical decision‐making and coordination with emergency systems. Technical and clinical competencies such as casualty care, clinical assessment and procedural skills ensure readiness for general and complex emergencies. Specialist competencies, including Hazmat response, CBR incidents and tactical care, prepare paramedics for specific high‐risk scenarios. Competency‐based education supports skill acquisition and consistency in disaster preparedness. Incorporating these competencies into training

**TABLE 2:** Results of content analysis of competencies required of paramedics in prehospital emergency care.

Sub‐categories	Categories	Main categories
Interpersonal competence in prehospital emergency care	Interpersonal competence in prehospital emergency care	Interpersonal and Teamwork Competence
Development of interpersonal skills in simulation‐based training		
Interprofessional interaction within resuscitation teams		
Teamwork and task management in prehospital emergency care	Teamwork competence in prehospital emergency care	
Teamwork competence in resuscitation situations		
Teamwork in the prehospital emergency care setting		
Interprofessional collaboration in prehospital emergency care		
Teamwork in urgent prehospital emergency situations		
Collaboration with other professional groups in prehospital emergency care		
Organizing operations in prehospital emergency situations		
Allocation of tasks and roles in prehospital emergency care		
Supporting family members and communication at the time of death	Communication competence in prehospital emergency care	
Learning communication skills through patient encounters		
Professional communication competence in prehospital emergency care		
Communication with patients and team members in acute situations		
Application of emotional skills in patient encounters	Paramedics’ interpersonal competence	
Empathic patient encounters and acknowledgement of emotions		
Negotiation skills and constructive dialogue in clinical situations		
Health counselling and patient guidance in prehospital emergency situations	Health counselling and patient guidance in prehospital emergency care	
Promoting holistic health perspectives in prehospital emergency care		
Early patient contact and health education in prehospital emergency care		
Leadership in prehospital resuscitation teams	Leadership competency in prehospital emergency care	Cognitive and Decision‐Making Competence
Operational leadership in resuscitation		
Leadership of prehospital emergency operations in acute situations		
Situational leadership in prehospital emergency care		
Managing decision‐making during resuscitation	Paramedics’ decision‐making competence	
Decision‐making ability across prehospital emergency situations		
Decision‐making in high‐acuity prehospital emergency situations		
Situation‐based decision‐making in prehospital emergency care		
Practice of problem‐solving in training situations	Problem‐solving competence in prehospital emergency care	
Problem‐solving capacity in prehospital emergency situations		
Problem‐solving skills in complex prehospital emergency situations		
Situational awareness of paramedics	Situational awareness competence in prehospital emergency care	
Management of the situational picture during resuscitation		
Management of the situational picture in prehospital emergency care		
Situational picture formation in evolving situations		
Situational anticipation in urgent prehospital emergency situations		
Maintaining attention and concentration	Paramedics’ cognitive competence	
Recall and cognitive reasoning		
Verbal expression and comprehension skills		
Professional commitment to the patient’s best interests	Paramedics’ professionalism	Professionalism and Ethical Competence
Professional ethical practice in prehospital emergency care		
Reflection and professional development	Paramedics’ self‐directed learning and professional growth	
Goal‐oriented competence development		
Management of safety risks in prehospital emergency situations	Paramedics’ occupational safety competence	
Patient safety risk management		
Competence in advanced clinical procedures in prehospital emergency care	Paramedics’ clinical competence in prehospital emergency care	Clinical competence
Advanced‐level competence in clinical procedures in prehospital emergency care		
Clinical competence across different age groups		
Management of advanced‐level clinical procedures in complex special situations		
Advanced‐level clinical competence in complex and special situations		
Recognition and care of children’s special needs in prehospital emergency care		
Assessment of care needs among older patients in prehospital emergency care		
Basic‐level clinical competence in prehospital emergency situations		
Application of clinical skills in complex and high‐demand situations		
Assessment of patient condition and provision of care in prehospital care		
Monitoring of critically ill patients in emergency medical services		
Initiation of resuscitation in an emergency medical situation		
Development of technical skills in simulated clinical settings		
Development of clinical skills in simulation‐based training		
Development of clinical competence through continuing education		
Maintenance of clinical professional competence		
Basic‐level readiness for prehospital emergency care	Professional competence in prehospital emergency care	Competence Across Paramedic Certification Levels and in Special or High‐Stress Situations
Key competence areas in prehospital emergency care		
Maintaining professional competence in prehospital emergency care		
Care of nonurgent ambulance missions		
Basic‐level competence in special situations in prehospital emergency care	Special situation competence in prehospital emergency care	
Advanced‐level professional competence in special situations		
Preparedness for humanitarian crises in prehospital emergency care		
Comprehensive competence in exceptional situations		
Preparedness for chemical, biological and radiological emergencies		
Tactical prehospital emergency care readiness		
Care of heat‐related illnesses in special situations		

### 2.7. Assessing Confidence in the Review Findings

The GRADE‐CERQual (Confidence in the Evidence from Reviews of Qualitative research) approach was used to assess confidence in the review findings [83]. Confidence in each of the five main competence domains was assessed across four GRADE‐CERQual components: methodological limitations, coherence, adequacy of data and relevance. The GRADE‐CERQual assessment was conducted by two reviewers. Any disagreements were resolved through discussion, and the final assessment was reached by consensus. Based on the overall assessment of these four components, the level of confidence in each main competence domain was rated as high, moderate, low or very low. Detailed GRADE‐CERQual assessments and the corresponding justifications are presented in Supporting File 4.

## 3. Results

The review articles included in this umbrella review were published between 2014 and 2023. The rationale for this time frame was to capture the long‐term development of research evidence on competence in out‐of‐hospital emergency medical care while ensuring that the material also reflects contemporary research. The final dataset comprised eight reviews originating from Australia [77, 80], Canada [78], Sweden [74], Saudi Arabia [73], New Zealand [76] and the United Kingdom [75, 79]. Of the included reviews, five were scoping reviews [73, 76–79], two were systematic reviews [75, 80] and one was an integrative review [74].

As a result of content analysis, the competence required in out‐of‐hospital emergency medical care was divided into five main categories. Based on the GRADE‐CERQual assessment, confidence in each of the five main categories was rated as moderate. Interpersonal and teamwork competence comprised interpersonal skills in prehospital emergency care, health counselling and patient guidance in prehospital emergency care, as well as teamwork skills and communication competence in prehospital emergency care. Cognitive and decision‐making competence included leadership competence in prehospital emergency care, paramedics’ cognitive skills, decision‐making competence, problem‐solving skills in prehospital emergency care, and situational awareness competence in prehospital emergency care. Professionalism and ethical competence consisted of paramedics’ professionalism, professional ethical practice in prehospital emergency care, self‐directed learning and professional growth, as well as paramedics’ occupational safety competence. Clinical competence included paramedics’ clinical competence in prehospital emergency care. Competence across paramedic certification levels and in special or high‐stress situations included professional competence in prehospital emergency care and special situation competence in prehospital emergency care (see Table 2).

### 3.1. Interpersonal and Teamwork Competence

This domain was present in six reviews. *Interpersonal competence in prehospital emergency care* was identified as a fundamental component of effective resuscitation team performance [79]. Simulation‐based training supported the development of these skills in a safe learning environment, and the use of responsive simulation manikins enhanced participants’ professional competence [74]. *Teamwork competence* was identified as a core component of nontechnical skills and enabled effective collaboration in patient care [78]. In resuscitation situations, teamwork supported coordinated action and enhanced patient safety [79]. Within interprofessional teams, teamwork facilitated collaboration between different professional groups [78]. Training in teamwork competence contributed to the development of nontechnical capabilities and increased novice paramedics’ readiness to function in demanding situations [80]. *Communication competence* supported teamwork, facilitated the understanding of patients’ needs and contributed to the development of trusting interactional relationships [78]. Communicating a patient’s death and supporting family members required particularly advanced communication competence [76].

Interaction occurring in patient encounters further strengthened communication capabilities and supported the continuous development of these skills [74]. *Paramedics’ interpersonal competence* was reflected in empathic patient encounters, recognition of patients’ emotions and the regulation of their own emotional responses in patient situations [78, 80]. Negotiation skills and constructive dialogue promoted mutual understanding and facilitated smooth collaboration in patient care [78]. *Health counselling and patient guidance focused* on motivating and supporting patients in adopting health‐promoting practices, strengthening holistic health perspectives and highlighting factors influencing health [75].

### 3.2. Cognitive and Decision‐Making Competence

This domain was present in three reviews. *Leadership competence* in prehospital emergency care enabled the guidance and support of team performance in acute situations. Situational leadership strengthened team effectiveness and supported the attainment of shared goals [78]. In resuscitation team performance, leadership was emphasised alongside communication and teamwork to ensure the smooth delivery of patient care [79]. *Decision-making competence* was grounded in situational assessment and in the collection and evaluation of accurate and relevant information, which informed the consideration of alternatives and the formulation of context‐specific and well‐justified decisions [78]. In resuscitation situations, effective decision‐making requires shared situational awareness, leadership competence and smooth collaboration within the team [79]. *Problem-solving competence* constituted a central area of competence in prehospital emergency care, as it enabled the management of complex situations and the making of justified decisions under demanding conditions [78]. Simulation‐based training enhanced problem‐solving capacity and strengthened readiness to apply learned skills in practical situations [74]. *Situational awareness competence* enabled the assessment, anticipation and management of situations and supported effective performance in rapidly changing conditions [78]. In resuscitation situations, shared situational awareness improved team performance and decision‐making, reduced cognitive load and promoted patient safety [79]. *Cognitive competence* enabled the reception, processing and clear articulation of information in prehospital emergency situations. Maintaining attention supported the identification of essential information and sustained focus on situationally relevant factors, while memory and reasoning abilities enabled the recall of critical information and guided action in dynamically changing conditions [78].

### 3.3. Professionalism and Ethical Competence

This domain was present in four reviews. *Paramedic professionalism* was shaped through continuous professional development, in which nontechnical skills complemented and supported technical competence. Values such as competence, compassion, commitment and ethical judgement strengthened professionalism, guided professional practice and supported holistic patient care [80]. *Ethical competence* was manifested in responsible practice and in decisions that promoted the patient’s best interests. In decision‐making, ethical judgement was particularly emphasised in situations where urgency, uncertainty or patients’ individual needs challenged professional values and principles of practice [80]. *Self-directedness and professional growth* were reflected in the readiness to critically evaluate one’s own practice and to develop competence in a goal‐oriented manner. Self‐assessment supported the development of these capacities, facilitated the identification of strengths and areas for improvement and promoted continuous professional learning [74]. *Occupational safety competence* was emphasised in demanding and emotionally challenging situations, in which environmental risk factors, such as strong emotional reactions from family members, could complicate decision‐making, for example, in relation to the termination of resuscitation [76]. Ensuring patient safety required the ability to identify, assess and manage risks in a manner that safeguarded both patients and paramedics [75].

### 3.4. Clinical Competence

This domain was identified in three reviews. *Paramedics’ clinical competence in prehospital emergency care* consisted of basic‐level clinical competence required across a wide range of prehospital emergency care assignments, as well as more advanced clinical competence needed in special situations and demanding prehospital emergency care assignments [77]. The assessment of the patient’s condition and the implementation of care required the ability to identify changes, make justified decisions and deliver appropriate care under variable conditions [75]. Advanced‐level competence included life‐saving interventions for critically ill or severely injured patients, assessment of the patient’s condition and care needs, continuous monitoring and resuscitation management [73, 77]. Operating in complex and special situations required advanced clinical expertise and the ability to apply acquired competence in varying and demanding circumstances [73, 77]. In the care of patients of different ages, clinical competence was grounded in readiness to recognise individual needs and adapt care accordingly. Paramedics required additional competences to care for children with special healthcare needs and to assess and manage older patients [75].

### 3.5. Competence Across Paramedic Certification Levels and in Special or High‐Stress Situations

This domain was identified in three reviews. The mastery of *professional competence* was grounded in capabilities through which theoretical knowledge and practical skills were integrated to support professional practice and development [77]. Professional competence encompassed clinical, ethical and interpersonal dimensions that were manifested as compassion, self‐confidence, ethical judgement and commitment in patient care as well as in interprofessional collaboration [80]. The management of minor injuries and the provision of home visits required the ability to apply this competence in nonurgent care situations and reinforced the role of prehospital care in supporting continuity across the care pathway [75]. *Special situation competence* reflected paramedics’ readiness to function in exceptional and demanding conditions. Basic‐level paramedics were required to demonstrate competence in managing major incidents and disasters, as well as readiness to collaborate with other agencies. Advanced‐level paramedics were required to demonstrate broad clinical competence and the ability to operate within multidisciplinary and rapidly changing operational environments. Chemical, biological and radiological threats, hazardous materials incidents, humanitarian crises and tactical emergency medical care additionally require supplementary specialist competence. Special situation competence also included the ability to address the needs of patients requiring additional support, as well as to provide psychological support to patients and their family members [77]. In the context of large public events, paramedics’ capacity to assess care needs and provide appropriate treatment reduced the burden on the healthcare system [75] (Figure 2).

**FIGURE 2:**
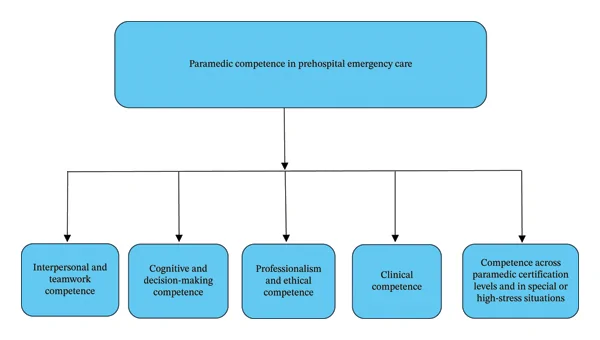
Framework of paramedic competence in prehospital emergency care identified in an umbrella review.

## 4. Discussion

Based on the findings of this umbrella review, paramedic competence in out‐of‐hospital emergency care can be conceptualised as a multidimensional and integrated framework comprising several interrelated domains. Rather than representing discrete or isolated areas of competence, these domains collectively underpin the delivery of safe, high‐quality and patient‐centred care in demanding and unpredictable prehospital environments.

Within this framework, *interpersonal competence* represents a central component of paramedic practice, particularly in patient encounters and emotionally demanding situations, where professional interaction supports patients’ experiences and the management of care situations [78, 80]. Empathic and patient‐centred interaction supports patients’ experiences and contributes to successful care encounters [84]. *Teamwork competence* constitutes a central component of paramedics’ nontechnical competence, as it enables high‐quality patient care and effective interprofessional collaboration, particularly in demanding prehospital situations [78]. Teamwork supports the appropriate distribution of tasks and responsibilities in complex prehospital contexts, thereby facilitating smooth operational performance and enhancing patient safety. Interpersonal interaction competence is a key prerequisite for the effective execution of prehospital tasks, as it supports the development of shared situational understanding and effective action across diverse prehospital scenarios [79]. Effective interaction strengthens team functioning and promotes both patient safety and quality of care [85]. *Communication competence* supports teamwork, facilitates understanding of the patient’s situation and strengthens the development of trust within care encounters [78]. The quality of communication is associated with positive care outcomes and patients’ perceived quality of care [86]. *Health counselling and patient education* enhance patients’ understanding of their own condition. They support engagement in self‐care, as evidenced by increased motivation and a more holistic approach to health [75]. The expansion of prehospital emergency care into health counselling and health information provision reflects changing healthcare needs and constrained healthcare resources [10, 30].

*Leadership competence* in prehospital emergency care supports team functioning and patient care, particularly in acute situations where coordination of actions and clarity of decision‐making are essential [78]. In prehospital emergency care, situational leadership is shaped by the demands of the operational environment and the objectives of the task. The complex and dynamic nature of prehospital emergency care requires leadership that supports operational control and smooth collaboration among multiple actors [87]. Effective *decision-making* requires shared situational awareness within the team, leadership competence and seamless collaboration, along with careful consideration of alternatives and the formulation of well‐justified decisions [78, 79]. Decision‐making in prehospital emergency care is not linear but dynamic and iterative, in which flexibility and the capacity to adapt to unforeseen changes are critical to successful outcomes [88]. *Problem-solving competence* is particularly central in demanding and rapidly evolving situations, as it supports the management of complex scenarios and enables logical and well‐founded decisions [78]. Simulation‐based training promotes the development of problem‐solving skills and facilitates the transfer of knowledge to practical situations [74]. Goal‐oriented training further supports the development of decision‐making and critical thinking skills [89]. *Situational awareness* strengthens team performance by reducing cognitive load, improving the quality of decision‐making and promoting shared situational understanding. It is a key factor in demanding and cognitively taxing prehospital emergency care situations, such as resuscitation tasks [78, 79]. *Cognitive competence* forms the foundation for decision‐making and situation‐appropriate action in prehospital emergency care [78]. These abilities are reflected in the ability to gather, interpret and anticipate information, as well as to direct attention to factors that are most relevant to the situation.

*Paramedic professionalism* is grounded in both clinical competence and broader professional capabilities. Commitment, compassion and ethical judgement structure professional practice and guide everyday work [80]. *Ethical competence* is manifested in responsible, patient‐centred decisions in situations where uncertainty, urgency and limited resources place particular demands on professional practice [80]. Ethical judgement is especially critical in uncertain, time‐pressured situations and in contexts characterised by constrained resources [90]. *Self-directedness and professional growth* are grounded in the ability to critically evaluate one’s own practice and to continuously develop competence [74]. The increasing complexity of the prehospital care environment and the expanding competence requirements render these capacities central to professional practice. *Occupational safety competence* is particularly important in demanding and emotionally taxing situations, as safe practice is based on the ability to identify and manage situational risks, which is a prerequisite for both patient and paramedic safety [75]. The increasing prevalence of violence in healthcare further highlights the importance of occupational safety competence, as physical violence or the threat thereof is more common in social and healthcare services than in other sectors [91].

*Clinical competence* is based on the integration of theoretical knowledge and practical skills across diverse prehospital care tasks. *Paramedics’ clinical competence in prehospital emergency care* is particularly evident in demanding and rapidly changing situations, where broad clinical skills and accumulated experience support safe and effective practice [73, 77]. Caring for patients across different age groups requires competence to be adapted to the patient’s age, functional capacity and individual needs. Repeated practical training and experience with different patient groups and prehospital care tasks support the development of clinical skills and their application across different situations [92].

*Professional competence* constitutes an integrated whole in which theoretical knowledge, technical skills, decision‐making abilities and broader professional capabilities are interconnected, providing a foundation for the quality of prehospital emergency care and effective collaboration among different actors [77]. Changes in the operational environment require continuous competence development and readiness to adapt professional roles as service needs evolve [10]. *Special situation competence* refers to the ability to apply paramedic expertise in exceptional and demanding conditions that require close and coordinated collaboration with other authorities. It also encompasses the ability to address the needs of patients requiring additional support and to provide psychological support to patients and their family members [77]. Interagency collaboration is a key element of successful disaster management; however, its effectiveness may be compromised by a lack of shared operational models, unclear responsibilities and communication breakdowns that hinder coordinated action [18, 93].

The competence domains identified in this review are largely consistent with international paramedic competence frameworks, supporting the view that the core competence requirements of out‐of‐hospital emergency care are largely shared across countries and healthcare systems. Although the review focused on the competences required by paramedics in out‐of‐hospital emergency care, not all the identified main competence domains are unique to paramedicine. Interpersonal and teamwork competence, cognitive and decision‐making competence, professionalism and ethical competence, and clinical competence describe competence domains that are also required in other healthcare professions. Competence across paramedic certification levels and in special or high‐stress situations was identified as a main competence domain particularly related to the paramedic context. Thus, the findings also overlap with other areas of healthcare, but the distinctive characteristics of out‐of‐hospital emergency care should be considered when interpreting and applying them.

## 5. Limitations and Strengths

This umbrella review focused on paramedics working in out‐of‐hospital emergency medical services, which may limit the generalisability of the findings to other healthcare settings. Only English‐ and Finnish‐language publications were included, which may have introduced language bias and excluded relevant evidence published in other languages. The synthesis was based on a limited number of reviews, which may have affected the comprehensiveness of the identified competence domains. The geographical representation of the included reviews was limited, as the reviews originated from only six countries. Emergency medical services systems, education, professional roles and scopes of practice vary across countries, which may influence the competences required in emergency medical services. Therefore, the international generalisability of the findings should be interpreted with caution.

Combining systematic, scoping and integrative reviews enabled the broad identification of competence domains but increased the methodological heterogeneity of the synthesis. The review types differ in their aims, research questions, inclusion criteria, critical appraisal practices and synthesis methods, and the evidence they provide is therefore not directly comparable. This may have affected the comparability of the findings and the interpretation of the synthesis. On the other hand, combining different review types enabled a broader examination of the research topic and provided a more comprehensive overall picture than the use of a single review type alone would have allowed.

The possibility of publication bias cannot be ruled out, as published reviews may not represent all the research evidence available on the topic. Consequently, the synthesis may have placed greater emphasis on competence domains for which published research evidence was available. Reporting bias may also have occurred in the included reviews if findings related to competence domains were selectively reported. This may have influenced which competence domains were identified and emphasised in this synthesis. Assessing the components that determine confidence in the review findings—methodological limitations, relevance, adequacy of data and coherence—requires judgement and may be open to interpretation.

The evidence included in the reviews addressed the competences required to care for older patients and children with special healthcare needs. In contrast, the reviews did not address the competences required to care for patients with mental health or substance use disorders.

This review has several strengths. To the best of our knowledge, it is the first umbrella review to systematically synthesise research evidence on the competences required of paramedics in out‐of‐hospital emergency medical services. A comprehensive search across multiple databases enabled the identification of relevant reviews. Adherence to JBI methodological guidance supported the consistent conduct of review selection, critical appraisal, data extraction and synthesis. We used the GRADE‐CERQual approach to assess confidence in the review findings. Adherence to the PRISMA 2020 checklist increased the transparency of reporting the search and selection process (Supporting File 3).

## 6. Conclusion

This umbrella review provides an overview of the competences required of paramedics in out‐of‐hospital emergency medical care, both now and in the future. The findings indicate that paramedic competence has expanded beyond the traditional management of emergency patients toward more autonomous and versatile professional practice, in which the assessment of care needs and the management of nonurgent patients have become increasingly important. At the same time, competence requirements increasingly involve broader interprofessional collaboration across organisational and sectoral boundaries.

In contrast to previous research, which has often examined paramedic competence in terms of individual skills or narrowly defined domains, this umbrella review synthesises existing evidence into a structured framework and highlights the interrelationships between different competence domains.

The findings emphasised the importance of key competence domains, including teamwork, communication, leadership, clinical competence and situational awareness. The limited reporting of some competence domains indicates a need for further research to clarify their significance and potential future competence requirements. In particular, further research is needed on medication competence and competence in the use of medical devices.

Based on the findings, clearer distinctions should be made between competences included in basic education and those requiring continuing professional development. Further research should also examine which educational and assessment methods most effectively support the maintenance and development of competence within a changing operational environment and an expanding scope of practice.

## Author Contributions

Marko Leinonen, MHSc: conceptualisation, methodology, formal analysis, investigation, writing–original draft, visualisation.

Petri Roivainen, PhD: conceptualisation, methodology, writing–review and editing.

Anna‐Maria Tuomikoski, PhD: writing–review and editing.

Merja Sjöman, MHSc: writing–review and editing.

Kristina Mikkonen, PhD, Professor: conceptualisation, methodology, formal analysis, visualisation, writing–review and editing, supervision.

## Funding

This research received no specific grant from any funding agency in the public, commercial or not‐for‐profit sectors.

## Conflicts of Interest

The authors declare no conflicts of interest.

## Supporting Information

Additional supporting information can be found online in the Supporting Information section.

## Supporting information


**Supporting Information 1** Supporting file 1: Search strategy used in electronic databases.

**Supporting Information 2** Supporting file 2: Quality appraisal of the included studies.

**Supporting Information 3** Supporting file 3: PRISMA 2020 checklist.

**Supporting Information 4** Supporting file 4: GRADE‐CERQual evidence profile.

## Data Availability

The data supporting the findings of this study are derived from previously published studies, which are cited within the article. All extracted data are included in this article and its supporting information.
