# Supplementary material for: Competences of Paramedics in Prehospital Emergency Care: An Umbrella Review
Source: Emerg Med Int. 2026 Nov 15;2026:5400734. doi: 10.1155/emmi/5400734 (PMC13770273; doi:10.1155/emmi/5400734)
Supplement: Supplementary file 1 [file EMMI-2026-5400734-s001.docx]

Supplementary file 1. Search strategy used in electronic databases

| **Database** | **Search strategy** |
| --- | --- |
| PubMed | ((("Paramedics"[Mesh] OR "medic" OR "emergency medical technician*" OR "ambulance clinician*" OR "emergency care practitioner*" OR "prehospital emergency nurse*" OR "paramedic nurse*" OR "emergency medical staff*" OR "ambulance staff*") AND ("emergency medical services"[Mesh] OR "prehospital emergency medicine"[Mesh] OR "prehospital care*" OR "emergency care provider" OR "out-of-hospital*") AND ( "professional competence"[Mesh] OR "competence*" OR "skill*" OR "knowledge*" OR "knowhow" OR "value*" OR "attitude*" OR "qualification*" OR "professional knowledge" OR "clinical competence" OR "clinical skills" OR "education")) |
| CINAHL | ((MH "Paramedics" OR "medic" OR "emergency medical technician*" OR "ambulance clinician*" OR "emergency care practitioner*" OR "prehospital emergency nurse*" OR "paramedic nurse*" OR "emergency medical staff*" OR "ambulance staff*") AND (MH "Emergency Medical Services" OR "prehospital emergency medicine" OR "prehospital care*" OR "emergency care provider" OR "out-of-hospital*") AND (MH "Professional Competence" OR "competence*" OR "skill*" OR "knowledge*" OR "knowhow" OR "value*" OR "attitude*" OR "qualification*" OR "professional knowledge" OR "clinical competence" OR "clinical skills" OR "education")) |
| Medic | ("paramedics" OR "medic" OR "emergency medical technician*" OR "ambulance clinician*" OR "emergency care practitioner*" OR "prehospital emergency nurse*" OR "paramedic nurse*" OR "emergency medical staff*" OR "ambulance staff*") AND ("emergency medical services" OR "prehospital emergency medicine" OR "prehospital care*" OR "emergency care provider" OR "out-of-hospital care") AND ("professional competence" OR "competence*" OR "skill*" OR "knowledge*" OR "know-how" OR "value*" OR "attitude*" OR "qualification*" OR "professional knowledge" OR "clinical competence" OR "clinical skills" OR "education") |
| Scopus | TITLE-ABS-KEY ( "medic" OR "emergency medical technician*" OR "ambulance clinician*" OR "emergency care practitioner*" OR "prehospital emergency nurse*" OR "paramedic nurse*" OR "emergency medical staff*" OR "ambulance staff*" ) AND TITLE-ABS-KEY ( "emergency medical services" OR "prehospital emergency medicine" OR "prehospital care*" OR "emergency care provider" OR "out-of-hospital*" ) AND TITLE-ABS-KEY ( "professional competence" OR "competence*" OR "skill*" OR "knowledge*" OR "knowhow" OR "value*" OR "attitude*" OR "qualification*" OR "professional knowledge" OR "clinical competence" OR "clinical skills" OR "education" )) |
| ProQuest | (((noft("Emergency Medical Technician*" OR "Advanced EMT*" OR "ambulance nurse*" OR "ambulance crew" OR "ambulance staff" OR "ambulance personnel" OR "Emergency care practitioner*" OR "Emergency medical staff" OR medic OR medics OR paramedic* OR "emergency nurse*") AND noft(Ambulance* OR "Emergency healthcare service*" OR "Emergency health care service*" OR "Emergency health service*" OR "Emergency medical service*" OR "Emergency service*" OR "emergency care" OR "Out-of-hospital" OR "out of hospital" OR Pre-hospital OR Prehospital OR "transportation of patient*" OR "patient transportation") AND noft(attitude* OR capabilit* OR competenc* OR education OR knowledge OR know-how OR Knowhow OR Learning OR Professional OR professionalism OR Qualification* OR Skill*) AND noft(review* OR overview* OR meta-analy* OR "meta analy*" OR "meta synthes*" OR meta-synthes* OR metareview* OR meta-review*)) OR ((noft("Emergency Medical Technician*" OR "Advanced EMT*" OR "ambulance nurse*" OR "ambulance crew" OR "ambulance staff" OR "ambulance personnel" OR "Emergency care practitioner*" OR "Emergency medical staff" OR medic OR medics OR paramedic* OR "emergency nurse*") AND noft(Ambulance* OR "Emergency healthcare service*" OR "Emergency health care service*" OR "Emergency health service*" OR "Emergency medical service*" OR "Emergency service*" OR "emergency care" OR "Out-of-hospital" OR "out of hospital" OR Pre-hospital OR Prehospital OR "transportation of patient*" OR "patient transportation") AND noft(attitude* OR capabilit* OR competenc* OR education OR knowledge OR know-how OR Knowhow OR Learning OR Professional OR professionalism OR Qualification* OR Skill*)) AND at.exact("Literature Review"))) AND (la.exact("ENG") AND stype.exact("Scholarly Journals") AND pd(20040101-20241231))) AND la.exact("English" OR "Finnish") AND pd(>20040101) |
