# Supplementary material for: Competences of Paramedics in Prehospital Emergency Care: An Umbrella Review
Source: Emerg Med Int. 2026 Nov 15;2026:5400734. doi: 10.1155/emmi/5400734 (PMC13770273; doi:10.1155/emmi/5400734)
Supplement: Supplementary file 2 [file EMMI-2026-5400734-s002.docx]

Supplementary file 2. Quality appraisal

| JBI Critical Appraisal Checklist for Systematic Reviews and Research Syntheses | Abelsson et al., 2016 | Alruwaili et al., 2019 | Andersson et al., 2019 | Cormack et al., 2020 | Evans et al., 2021 | Evans et al., 2014 | Gent 2016 | Horrocks et al., 2019 |
| --- | --- | --- | --- | --- | --- | --- | --- | --- |
| 1. Is the review question clearly and explicitly stated? | Yes | Yes | Unclear | Yes | Yes | Yes | Yes | Yes |
| 2. Were the inclusion criteria appropriate for the review question? | Yes | Yes | Yes | Yes | Unclear | Yes | Yes | Yes |
| 3.Was the search strategy appropriate? | Yes | Yes | Yes | Yes | Unclear | No | Yes | Yes |
| 4. Were the sources and resources used to search for studies adequate? | Yes | Yes | Yes | Yes | Yes | Yes | Unclear | Yes |
| 5. Were the criteria for appraising studies appropriate? | Yes | Yes | Yes | No | Yes | Yes | No | Not applicable |
| 6.Was critical appraisal conducted by two or more reviewers independently? | Yes | Yes | Yes | No | Yes | Yes | No | No |
| 7. Were there methods to minimize errors in data extraction? | Yes | Yes | No | No | Yes | Yes | No | Yes |
| 8. Were the methods used to combine studies appropriate? | Yes | Yes | Yes | No | Yes | No | No | Yes |
| 9. Was the likelihood of publication bias assessed? | Unclear | Yes | No | No | Unclear | No | No | Yes |
| 10.Were recommendations for policy and/or practice supported by the reported data? | Yes | Yes | Yes | Yes | Yes | Yes | Yes | Yes |
| 11.Were the specific directives for new research appropriate? | Yes | Yes | Yes | Yes | Yes | Yes | Yes | No |
| Score | 10/11 | 11/11 | 8/11 | 6/11 | 8/11 | 8/11 | 5/11 | 8/11 |
