# Supplementary material for: Competences of Paramedics in Prehospital Emergency Care: An Umbrella Review
Source: Emerg Med Int. 2026 Nov 15;2026:5400734. doi: 10.1155/emmi/5400734 (PMC13770273; doi:10.1155/emmi/5400734)
Supplement: Supplementary file 4 [file EMMI-2026-5400734-s004.docx]

Supplementary file 4. GRADE-CERQual evidence profile

| **Summary of review finding** | **Studies contributing to the review finding** | **Methodological limitations** | **Coherence** | **Adequacy** | **Relevance** | **CERQual assessment of confidence in the evidence** | **Explanation of CERQual assessment** |
| --- | --- | --- | --- | --- | --- | --- | --- |
| Interpersonal and teamwork competence comprised interpersonal competence in prehospital emergency care, teamwork competence, communication competence, paramedics’ interpersonal competence, and health counselling and patient guidance. | Abelsson et al., 2016  Anderson et al., 2019  Cormack et al., 2020  Evans et al., 2014  Evans et al., 2021  Gent, 2016 | Minor methodological limitations. (1 review with minor, 3 reviews with moderate methodological limitations (limitations related to the review question or inclusion criteria, search strategy, data extraction, synthesis methods, and assessment of publication bias), and 2 reviews with serious methodological limitations (limitations related to the adequacy of search sources and resources, critical appraisal criteria, independent critical appraisal, data extraction, synthesis methods, and assessment of publication bias)) | No or very minor concerns about coherence | Minor concerns about adequacy. The finding was supported by 6 contributing reviews, providing sufficient data across several aspects of interpersonal and teamwork competence. | No or very minor concerns regarding relevance. The contributing reviews were relevant to the review question, as they addressed paramedics and competence in prehospital emergency care. | Moderate confidence | 6 reviews with minor methodological limitations overall. No or very minor concerns about coherence and relevance. Minor concerns about adequacy. |
| Cognitive and decision-making competence encompassed leadership, decision-making, problem-solving, situational awareness, and the ability to process and apply information in rapidly changing prehospital situations. | Abelsson et al., 2016  Cormack et al., 2020  Evans et al., 2021 | Minor methodological limitations. (1 review with minor, 1 review with moderate methodological limitations (limitations related to the inclusion criteria, search strategy, and assessment of publication bias), and 1 review with serious methodological limitations (limitations related to critical appraisal criteria, independent critical appraisal, data extraction, synthesis methods, and assessment of publication bias)). | No or very minor concerns about coherence | Minor concerns about adequacy.  The finding was supported by 3 contributing reviews, which provided sufficient data on different aspects of cognitive and decision-making competence. | No or very minor concerns about relevance. The contributing reviews addressed the review question, as they examined paramedics and the competence required in out-of-hospital emergency medical care. | Moderate confidence | 3 reviews with minor methodological limitations overall. No or very minor concerns about coherence and relevance. Minor concerns about adequacy. |
| Professionalism and ethical competence comprised paramedic professionalism, ethical competence, self-directedness and professional growth, and occupational safety competence. | Abelsson et al., 2016  Anderson et al., 2019  Evans et al., 2014  Gent, 2016 | Moderate methodological limitations. (1 review with minor, 2 reviews with moderate methodological limitations (limitations across the two reviews related to the review question, search strategy, data extraction, synthesis methods, and assessment of publication bias), and 1 review with serious methodological limitations (limitations related to the adequacy of search sources and resources, critical appraisal criteria, independent critical appraisal, data extraction, synthesis methods, and assessment of publication bias)). | No or very minor concerns about coherence | Minor concerns about adequacy. The finding was supported by 4 contributing reviews, which provided sufficient data on different aspects of professionalism and ethical competence. | No or very minor concerns about relevance. The contributing reviews addressed the review question, as they examined paramedics and the competence required in out-of-hospital emergency medical care. | Moderate confidence | 4 reviews with moderate methodological limitations overall. No or very minor concerns about coherence and relevance. Minor concerns about adequacy. |
| Clinical competence comprised paramedics’ clinical competence in prehospital emergency care. | Alruwaili et al., 2023; Evans et al., 2014; Horrocks et al., 2019**.** | Minor methodological limitations. (1 review with no or very minor methodological limitations and 2 reviews with moderate methodological limitations (limitations related to the search strategy, independent critical appraisal, methods used to combine studies, assessment of publication bias, and specific directives for new research)). | No or very minor concerns about coherence | Minor concerns about adequacy. The finding was supported by 3 contributing reviews, which provided sufficient data on clinical competence in out-of-hospital emergency medical care. | No or very minor concerns about relevance. The contributing reviews addressed clinical competence required in out-of-hospital emergency medical care. | Moderate confidence | 3 reviews with minor methodological limitations overall. No or very minor concerns about coherence and relevance. Minor concerns about adequacy. |
| Competence across paramedic certification levels and in special or high-stress situations comprised professional competence and special situation competence in prehospital emergency care. | Evans et al., 2014; Gent, 2016; Horrocks et al., 2019 | Moderate methodological limitations (2 reviews with moderate methodological limitations (limitations related to the search strategy, independent critical appraisal, methods used to combine studies, assessment of publication bias, and specific directives for new research), and 1 review with serious methodological limitations (limitations related to the adequacy of search sources and resources, critical appraisal criteria, independent critical appraisal, data extraction, methods used to combine studies, and assessment of publication bias)). | No or very minor concerns about coherence | Minor concerns about adequacy. The finding was supported by 3 contributing reviews, which provided sufficient data on professional competence and special situation competence in out-of-hospital emergency medical care. | No or very minor concerns about relevance. The contributing reviews addressed the competence required across different paramedic certification levels and special situation competence in out-of-hospital emergency medical care. | Moderate confidence | 3 reviews with moderate methodological limitations overall. No or very minor concerns about coherence and relevance. Minor concerns about adequacy. |
